# Bidirectional Associations across Time between Entitativity, Positive Affect, Generosity, and Religiousness in Adolescents Training with a Religiously Affiliated Charity Marathon Team

**DOI:** 10.3390/ijerph17030686

**Published:** 2020-01-21

**Authors:** Sarah Schnitker, Jennifer Shubert, Benjamin Houltberg, Nathaniel Fernandez

**Affiliations:** 1 Psychology and Neuroscience, Baylor University, Waco, TX 76798, USA; Jennifer_Shubert@baylor.edu; 2 Performance Science Institute, University of Southern California, Los Angeles, CA 90007, USA; houltber@marshall.usc.edu; 3 University Counseling Center, Azusa Pacific University, Azusa, CA 91702, USA; nathanielfernandezfuller@gmail.com

**Keywords:** adolescents, sports, marathon training, fundraising, generosity, religiosity, positive affect, entitativity

## Abstract

Numerous studies have established that participation in regular physical activity provides physical, cognitive, and affective benefits to adolescents, but fewer studies have examined how athletic involvement might affect character, social, or religious developmental markers of psychosocial functioning. The purpose of this study is to examine the bidirectional associations between entitativity (group closeness), positive affect, generosity, and religiousness across time among adolescents and emerging adults involved in charitable marathon training. We collected data from 396 adolescents and emerging adults who trained for half/full marathons with a religiously affiliated charity team. Participants completed measures at three occasions over 18 weeks (pre-training, mid-training, post-race). We conducted cross-lagged path analysis of latent factors to study concurrent and longitudinal effects of intrinsic religiousness, positive affectivity, and entitativity on interpersonal generosity and fundraising. Participants who reported higher levels of pre-training generosity were more likely to experience positive affect during training, which predicted higher levels of post-race generosity. Likewise, the internalization of religious ideas, reflected in increased intrinsic religiousness during training, was associated with higher post-race generosity. Overall, results support the potential of charitable sporting events to promote positive psychosocial developmental outcomes.

## 1. Introduction

Sports are often presented as ‘character building’ activities in western culture (e.g., as narrated through films such as *Chariots of Fire*). Indeed, numerous studies have shown sports participation not only provides physical cognitive, and affective benefits to adolescent [1,2] but also is associated with a range of positive outcomes including prosociality and character strengths in adolescents [3]. Yet, work showing decrements in character strengths and morality in some sporting contexts [4,5,6] suggest that sports are highly contextualized, highlighting the need for greater specificity in examining outcomes of sport involvement. Although not representative of all types of sports in which young people participate, training for charitable fitness events, including half or full marathons, is increasing in popularity among adolescents [7]. Charitable training organizations regularly present anecdotal evidence that people involved with a training team experience personal transformation through training (e.g., stories of runners on charitable training websites) [8]. Given that charitable marathon training may be especially well-suited to promoting the moral formation and well-being of young people, the purpose of this study is to examine the bidirectional associations between entitativity, positive affect, generosity, and religiousness across time among adolescents and emerging adults involved in charitable marathon training.

### 1.1. Charitable Marathon Training Context

Charitable training events provide an asset-rich context necessary for socioemotional and character development through the presence of mentors, role models, skill-building opportunities, and leadership roles [9]. Many of the organizations that recruit and motivate young people to participate in charitable training are explicitly religious (e.g., Team World Vision, Life Water International, Catholic Charities USA, Samaritan’s Purse) and imbue training activities with spiritual meaning and a focus on beyond-the-self ideals, offering opportunities for strengthening generosity and religiosity throughout the experience. Likewise, asset-rich contexts like charity training events promote multiple psychological indicators of well-being, such as positive affect during training, group bonding or sense of community, and general satisfaction from the physical accomplishment [10,11,12]. Although past work suggests charitable marathon training offers opportunities for enhancing psychosocial and moral development in young people, few studies have taken a process-based perspective in order to more fully elucidate how developmental processes unfold across time. We endeavor to examine the bidirectional associations not only among commonly studied socioemotional factors (e.g., positive affectivity and entitativity) but also among the less often studied moral-religious factors of generosity and connecting religiousness to identity. Analysis of bidirectional associations among these variables in an asset-rich setting will allow us to understand how the ways youth engage resources correspond to their development.

### 1.2. Generosity

Athletic teams—like charitable marathon training groups—that are connected to a beyond-the-self, transcendent, or religious causes are potentially powerful contexts for generosity formation [13]. Generosity is the propensity of a person to give objective (e.g., money, labor, time) or subjective (e.g., attention, emotions, energy) resources with the goal of increasing the good of others [14,15]. People can display generosity across the life course starting in childhood [16], but it is only during adolescence and emerging adulthood that people first begin to experience the autonomy that allows for substantive expression of generosity as well as refinement of requisite cognitive and emotional capacities (e.g., perspective taking, self-understanding, problem solving) [17] that allow for the full elaboration of character strengths, like generosity [18]. Not only is generosity an indicator of positive adjustment during adolescence and emerging adulthood, but it also launches a trajectory of generativity that may continue across the life course [19]. Previous research has demonstrated that changes in generosity are predicted by initial levels of prosocial and spiritual (but not health) motivations among adolescents training for charitable marathons, and changes in generosity are also predicted by changes in prosocial motivations [20].

### 1.3. Positive Affect

Previous research suggests aerobic exercise may lead to subsequent increases in positive affect [21] and that high activation of positive affect may lead to subsequent increases in exercise frequency [22]. Moreover, contexts that activate frequent and intense positive affective experience may also facilitate generosity development. Consistent with Fredrickson’s broaden-and-build theory [23], which identifies positive affect and emotions as provoking increases in openness and exploration of new possibilities in the environment, experimental evidence shows that inducing positive affect leads to increases in generous giving [24]. As people become more open to others in their environment when experiencing positive affect, they may be more likely to approach those who are different and give generously to those in need—even to those outside their group. For example, Johnson and Fredrickson [25] found that inducing positive affect eliminated White participants same-race facial bias such that they were able to recognize Black faces equally well as White faces. This openness creates a self-perpetuating cycle of positive affect and generosity. As people experience positive affect, they will become more generous, which produces additional positive affect [26]. Acts of generosity and volunteerism in adolescence and adulthood correlate with greater well-being across cultures [27,28], and neuroimaging studies show giving to others activates similar neural networks as receiving a reward [29]. Thus, positive affect and generosity are likely to show reciprocal positive associations across time among adolescents engage in charitable sporting contexts.

### 1.4. Entitativity

Entitativity is a feeling of belongingness to a group and the perception that the group is a cohesive entity [30]. Charitable marathon training teams tend to become meaningful social groups for runners, and runners may perceive these groups as more or less entitative. The extent to which adolescents and emerging adults feel a sense of entitativity is likely to affect the development of prosocial traits, though the effects of belonging will differ based on the group norms. According to costly signaling theory [31], high group cohesion (including entitativity) motivates people to demonstrate their commitment to the group even if it requires engaging in behavior that has a high personal cost (e.g., personal sacrifice involved in performing acts of generosity). People also tend to adapt their own giving behaviors to the group norm [32,33].

Therefore, membership in a highly entitative group that values generosity—like a charitable training team—may incentivize further generous acts by reinforcing a sense of group membership. In contrast, if group norms do not value generosity, highly entitative groups may inhibit the development of generosity. However, the associations between entitativity and prosocial behavior are complex and depend to a large extent on the makeup of a particular social network [34]. Finally, entitativity is likely related to participants’ positive affectivity. People who feel a sense of group belonging have more positive affect, and people with more positive affect are more likely to feel a sense of belonging [35,36].

### 1.5. Connecting Religiousness to Identity

Many charitable training teams are religiously affiliated, and participation in spiritual and religious contexts is a well-documented correlate of both financial and behavioral generosity [37,38,39]. Studies demonstrate associations between religiousness and generosity (as assessed by self-reports, peer-reports, and behaviors in the laboratory) [40,41]. However, little is known about the differing ways young people’s engagement in religiously affiliated athletic contexts support or hinder the development of generosity.

Many young people in the United States are exposed to religious ideas in childhood and adolescence, and, despite a decline in traditional religious participation, the majority of adolescents attend religious services at least periodically [42]. Explicitly religious contexts, like many charitable marathon training groups, provide adolescents and emerging adults the opportunity to internalize their religiousness in such a way that it serves as a resource for positive development by providing a shared vision, coherent life narrative, and meaning system [43]. However, the degree to which religious beliefs and attitudes are internalized to become a component of identity—forming a sense of intrinsic religiousness—differs across individuals. For this reason, researchers in psychology of religion distinguish between intrinsic and extrinsic forms of religiousness [44,45]. Whereas extrinsic religiousness involves using religion for instrumental ends, intrinsic religiousness involves incorporating religion into identity as an internalized value. Adolescents who participate in religious contexts are more likely to develop intrinsic religiousness that serves as a developmental asset than non-participators [46], and indeed, intrinsic religiousness tends to consistently predict prosocial development across cultures and contexts [47,48,49]. Thus, adolescents who internalize religious ideas in a religiously affiliated charitable training context will likely report high levels of intrinsic religiousness that will lead to subsequent high levels of reported generosity.

### 1.6. Current Study

In the present study, we survey adolescents and emerging adults as they train with Team World Vision (https://www.teamworldvision.org/), a religiously affiliated philanthropy. We employ a primarily exploratory approach to analyses. The marathon training process provides a meaningful temporal sequence for examining interrelations among personality constructs from the beginning of training to post-race. Following Lerner’s suggestions for Relational Developmental Systems research [9], we examine intrinsic religiousness, interpersonal generosity, positive affect, and entitativity in a cross-lagged path model to test for associations between these variables across three measurement occasions from pre-training to post-race. Relational Developmental Systems theory maintains that not only do the assets provided to adolescents predict adolescent thriving [50] but also the ways adolescents engage contexts matter for psychosocial and moral development [9]. For example, adolescents with higher levels of self-regulatory abilities benefitted more from extracurricular activity involvement than those with lower regulatory abilities in terms of positive youth development outcomes [51]. This approach allows examination of the patterns of influence of the variables across time and is useful in exploring initial patterns of effects where there is limited empirical support [52]. We test for all directional cross-lagged paths between the study variables across measurement occasions and specify autoregressive paths for all constructs across measurement occasions. We explore whether there are gender and/or age differences in our model, but the extant research does not support specific hypotheses.

Charitable sporting contexts provide a unique research opportunity to examine the behavioral expression of generosity in the form of fundraising behavior in addition to self-reported interpersonal generosity. Few previous studies have examined fundraising to assess generosity. However, in adolescents and emerging adults who have limited financial resources to donate, fundraising activity and other forms of social participation may be more typical modes of behavioral generosity [53]. Although a variety of factors may influence how much money people raise for charity [54], the money raised is—at least in part—likely dependent on the amount of effort and time spent fundraising. Thus, we hypothesize that the amount of funds raised may serve as a proxy of behavioral generosity and is included in our model testing. We will control for perceived levels of family wealth due to the possibility that participants may vary in access to people to solicit sponsorships for raising funds. However, given that fundraising has not been previously used to assess generosity, it is unknown whether funds raised will correlate with self-reported interpersonal generosity.

## 2. Method

### 2.1. Participants

We collected data from 396 adolescents and emerging adults (ages 12–22 years, *M* = 18.42, *SD* = 2.03) recruited from the Team World Vision (TWV) marathon running training teams. Participants were 37% male and 63% female. Participants came from diverse backgrounds: 61% Caucasian, 17% Latino/a, 10% African/African American, 6% Asian/Asian American, and 6% other ethnic groups. Participants also self-identified their family wealth as 0.8% very poor, 5.4% poor, 23.2% lower middle-class, 48.7% middle-class, 19.6% upper middle-class, and 2.3% upper-class/rich. The majority of participants, 88%, identified with a Christian religious tradition (primarily Protestants with 12 people identifying as Catholic).

An array of challenges and obstacles, including injury, lack of training due to the time commitment, exhaustion, and motivation, makes training and completing marathons difficult [55]. Of the 396 participants in the study, only 227 (57%) provided data at all three waves. Specifically, out of the 396 participants that completed surveys at time 1, 248 participants completed measures at time 2 and 229 completed measures at time 3. However, those with complete data did not differ from those with missing data at one or more waves on gender, socioeconomic status, or pre-training levels of the positive affectivity, intrinsic religiosity, group entitativity and interpersonal generosity when compared using *t*-tests. We did not exclude any participants from analyses.

### 2.2. Measures

Participants completed self-report measures of religion, positive affect, group connection, and generosity. Additionally, we used the amount of money raised by the participants as a behavioral measure of generosity. We collected additional measures from participants in addition to those used for present analyses, and full descriptions of all measures are provided in the online Supplemental Materials (File S2: Complete List of Measures).

#### 2.2.1. Group Entitativity/Closeness

Participants completed the Group Entitativity Measure (GEM) [56], which assesses perceived interconnections between self and others and has previously been used with adolescent samples [57]. Participants were asked to select one picture out of several options presented representing the relationship between the self and others (pictorially represented by overlapping circles) within the context of a particular group (i.e., the training team in the present study). The first picture displayed the greatest distance between self and others, whereas the last picture showed almost no distance between self and others so that the group and self are nearly overlapping. We coded the pictures with scores from 1 to 3, indicating low overlap to high overlap, respectively.

#### 2.2.2. Positive Affectivity

Participants completed the shortened version of the Positive and Negative Affect Scale for Children (PANAS-C) [58,59], which assessed positive affectivity. Participants rated themselves on a scale of 1 (very slightly or not at all) to 5 (extremely) for feelings of being joyful, cheerful, happy, lively, and proud. We computed a mean score of the five positive affect items. Cronbach’s α was 0.89, 0.89, and 0.87 at the three respective timepoints.

#### 2.2.3. Interpersonal Generosity

Participants completed the 10-item Interpersonal Generosity scale [15], which is a single-factor scale that measures six identifiable dimensions of interpersonal generosity (attention, compassion, open-handedness, self-extension, courage, and verbal expression). The scale has previously been used with adolescents [20]. Participants rated themselves on a 1 (Strongly Disagree) to 6 (Strongly Agree) scale for items such as “When it comes to my personal relationships with others, I am a very generous person” (open-handedness) and “My decisions are often based on concern for the welfare of others” (self-extension). We computed a mean score for the 10-items. Cronbach’s α was 0.88, 0.91, and 0.92 at the three respective timepoints.

#### 2.2.4. Fundraising amount Raised

TWV provided the amount of total donations raised in U.S. dollars for each runner, which we hypothesize might serve as a behavioral indicator of generosity. All participants were expected to engage in fundraising efforts throughout their training up until the race, but they were not penalized if they failed to raise funds. Each participant set up a donation page on TWV’s website, but fundraising efforts included both offline (e.g., canvassing the community in person, car washes, bake sales) and online activities (e.g., spreading the word about the donation opportunity through social media). Of note, the fundraising amount indicates successful efforts to secure money from others toward the fundraising goals of TWV rather than direct giving from the participants. Fundraising amounts ranged from $0 to $3092. To ensure fundraising had a similar variance structure as other variables in the model, fundraising was recoded using a quartile split where 0 = $0, 1 = $1–$99, 2 = $100–$449, and 3 = $450 or more for structural equation modeling analyses. Given the high likelihood that socioeconomic status (SES) affects participants’ access to sponsorship money, we controlled for SES in analyses using the fundraising variable.

#### 2.2.5. Duke University Religion Index

Participants completed the intrinsic religiousness subscale (three items) of the Duke University Religion Index (DUREL) [60], which includes three items: “In my life, I experience the presence of the Divine (i.e., God)”, “My religious beliefs are what really lie behind my whole approach to life”, and “I try hard to carry my religion over into all other dealings in life”. Participants rated items on a five-point scale from 1 (Definitely not true) to 5 (Definitely true of me), and we computed a mean score of the three items. Cronbach’s α was 0.90, 0.92, and 0.91 at the three respective timepoints. These items have been previously used with adolescents [61,62].

#### 2.2.6. Demographics

Participants reported their gender, age (in years), and school status (secondary school or college/university). Participants reported on how they would describe the wealth of their family based on the following categories: 1 = very poor, 2 = poor, 3 = lower middle-class, 4 = middle class, 5 = upper middle-class, and 6 = upper-class/rich. We used this variable of perceived family wealth as a proxy to assess socioeconomic status, which was used as a control in our model. The variable was treated as a continuous variable, as is typical in the developmental psychology literature, because M*plus* will only accept continuous endogenous variables in structural models.

### 2.3. Procedure

We obtained approval for all research activities from the Institutional Review Board. TWV personnel recruited adolescent and young adult participants from TWV running teams in Los Angeles and Chicago. Most participants in Chicago were recruited from a public high school and a private college May–June 2015 to run in the Chicago Marathon on 11 October 2015. Los Angeles participants were recruited from a few public and private secondary school teams and Christian church youth groups September–October 2015 to run in the LA Marathon on 14 February 2016. Given the difficulties of recruiting participants in this setting and lack of clear a priori effect sizes, we recruited as many participants as possible among participants involved in TWV training groups in Los Angeles and Chicago during our recruitment windows.

TWV leaders presented information about the study at recruitment meetings that were held at schools and churches, and people were given the opportunity to be contacted with further information. The study team then contacted potential participants and parents of minor potential participants to attain informed assent/consent. Participants then completed online questionnaires via Qualtrics at three time-points: Pre-Training (T1; 0 weeks), Mid-Training (T2; ≈15 weeks), and One Week Post-Marathon (T3; ≈18 weeks). The questionnaires included questions related to demographics and research questions of interest. The T2 questionnaire was administered after the longest training run—rather than at the training midpoint—because our practitioner partners described this as a key milestone in the training sequence and an important measurement point.

Participants engaged in weekly training sessions with their team and were provided training schedules to follow under the supervision of trained TWV captains. Additionally, TWV leaders presented motivational stories, information about fundraising, and spiritual messages during weekly team meetings. TWV communicated expectations to engage in fundraising to participants, but TWV did not penalize participants who failed to fundraise. Participants were compensated by the payment of their race entry fees (range from $120 to $180, depending on the city and the distance of the race).

The dataset is not shared on a public repository because IRB approval and parental consent were not obtained to publicly post information collected from minors. Moreover, identities of runners, their gender, and their race times are publicly available online for the Chicago and Los Angeles marathons, which threatens confidentiality. The dataset is available upon request from the corresponding author.

Findings from this dataset reporting bivariate latent growth curves models relating motivations (spiritual, prosocial, health) to the development of three virtues (self-control, patience, and interpersonal generosity) have been previously published [20]. In addition, a manuscript analyzing these data is under review proposing a multi-factor structure for self-control that predicts race completion and race times [63]. Finally, 24 participants were selected from the current dataset to complete in-person interviews related to identity development; a manuscript describing thematic analysis of these qualitative data is currently under review [64].

### 2.4. Analytic Approach

Initially, we tested longitudinal measurement invariance of intrinsic religiousness, positive affect, group entitativity, and interpersonal generosity to ensure measures were equivalent across time. We then used structural equation modeling in M*plus* [65] to examine longitudinal and cross-lagged associations in intrinsic religiousness, positive affect, group entitativity, and interpersonal generosity. We employed full information maximum likelihood (FIML) to estimate missing data and robust maximum likelihood for estimation, which relaxes the assumption of normal distributions in the data and all model comparisons included the Satorra–Bentler scaled Δχ^2^ test [66]. FIML is currently the most recommended approach to handling missing data, particularly in longitudinal studies with high attrition, such as ours [67]. Perceived wealth, as a proxy for SES, was entered as a control variable. Fundraising from time 3 was included in final model, and paths were specified from T2 substantive variables as well as control variables to T3 fundraising. Substantive variables were allowed to covary within waves and correlated residuals were estimated among the same indicators across waves to improve model fit. Although many cross-lag models constrain paths to be equal across measurement occasions, we allowed our cross-lags to vary because of the unique sequencing of the timepoints in the study design. We anticipated that effects from pre-training to mid-training might differ from those found mid-training to post-race. Model fit was determined via the Comparative Fit Index (CFI; with values ≥ 0.95 indicating good fit and values ≥ 0.90 indicating acceptable fit), and the Root Mean Squared Error of Approximation (RMSEA; with values ≤ 0.06 being acceptable) [68].

After estimating the full model, multigroup modeling was used to examine whether cross-lagged associations differed by age group or sex in an exploratory manner. For age we split the data based on whether the participants attended secondary school (*n* = 137, *M_age_* = 16.27 years, *SD* = 0.96) or college/university (*n* = 232, *M_age_* = 19.76 years, *SD* = 1.08). Measurement invariance across groups was tested using ΔCFI of 0.01 to ensure constructs were measured equivalently across age groups and across gender. Next, a cross-lagged model with equality constraints on structural paths was compared to the final scalar invariant model in which paths were free to vary across groups. Syntax for all analyses is available in File S1: Syntax for Autoregressive Models.

## 3. Results

Descriptive statistics bivariate correlations are presented in Table 1. (*p*-value cut-offs are displayed rather than exact values to improve readability). We observed strong, significant correlations within each variable across the three time points. Intrinsic religiousness, positive affectivity, and interpersonal generosity were all significantly and bidirectionally correlated across time points. Group entitativity at T2 was bidirectionally and positively correlated with positive affectivity at T2 and T3 only. 

### 3.1. Longitudinal Invariance Testing

As a first step, longitudinal measurement invariance was examined to ensure measures were equivalent across time (see Table 2). For positive affectivity and interpersonal generosity, item parcels were used as indicators of each latent factor. Little [69] recommends that all latent variables should be defined by a just-identified measurement model (i.e., three indicators per construct) and for this reason, we used item parcels for both positive affectivity and interpersonal generosity. Following recommendations by Little and colleagues [70], parcels were created using the balancing technique parcels high and low loadings. Invariance was established using change in CFI values; values greater than 0.01 were used to indicate worse fitting models [71]. Examinations of invariance by time indicated metric invariance (i.e., factor loading invariance) was achieved, based on non-significant differences in factor loadings over time (ΔCFI = 0.001) as was scalar invariance (i.e., intercept invariance; ΔCFI = 0.002; see Table 2).

### 3.2. Full Model with All Autoregressive and Cross-Lagged Pathways

**Figure 1:**
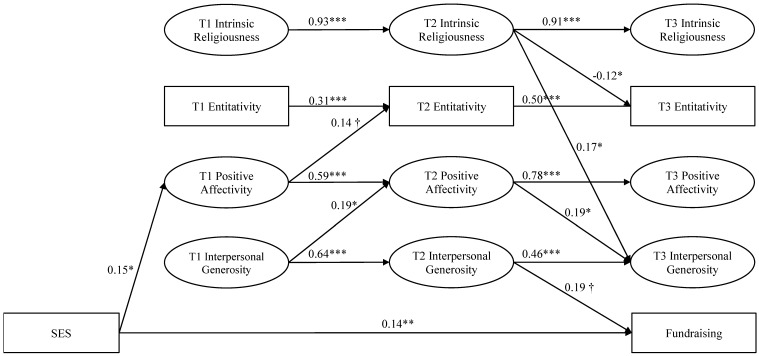
The effects of intrinsic religiousness, group entitativity, and positive affectivity on interpersonal generosity and fundraising across pre-training (T1), mid-training (T2), and post-race (T3). Note. Standardized path coefficients are displayed. For ease of presentation, only significant paths are shown. † *p* = 0.05, * *p* < 0.05, ** *p* < 0.01, *** *p* < 0.001.

The structural equation model demonstrated a good fit to the data, χ^2^ (285) = 366.27, *p* < 0.001, RMSEA = 0.03 (90% CI = 0.02 to 0.03), CFI = 0.98, TLI = 0.98. Figure 1 displays the significant path coefficients for the model; non-significant paths were included in the final model even though they are not displayed (i.e., no model trimming was used). Path coefficients and exact *p*-values for all paths specified are available in supplementary Table S1 in the online supplementary materials, but confidence intervals are not reported because they are not available in M*plus* when using full information maximum likelihood estimation with robust standard errors. The autoregressive path coefficients were significant across all three time points, suggesting high stability in the constructs across time. Concurrently, several variables significantly co-varied. Specifically, entitativity was positively correlated with positive affect at T3 and T2 but not T1. Positive affect at T3 was positively associated with interpersonal generosity at T3.

For the cross-lagged pathways, high levels of interpersonal generosity at T1 were associated with high levels of T2 positive affectivity and high levels of T2 positive affectivity were associated with high levels of T3 interpersonal generosity. T2 intrinsic religiosity was associated with high levels of T3 interpersonal generosity as well, but also, surprisingly, it was associated with low levels of entitativity. There were also two cross-lagged pathways that were approaching significance. Positive affectivity at T1 to T2 entitativity and T2 interpersonal generosity to fundraising totals. High levels of perceived family wealth as the control variable was only related to high levels of T1 positive affectivity and higher fundraising totals. Altogether, the model explained 86% of the variance in intrinsic religiousness at T2 and T3, 13% and 32% of the variance in entitativity at T2 and T3, 40% and 62% of the variance in positive affect at T2 and T3, 42% and 32% of the variance in interpersonal generosity at T2 and T3, and 8% of the variance in fundraising at T3.

### 3.3. Testing for Group Differences

A series of multigroup models examined whether cross-lagged paths differed by sex or age. For both groups, metric invariance (i.e., factor loading invariance) was achieved as was scalar invariance (i.e., intercept invariance). Next, for each model, we constrained cross-lagged structural paths to be equal across groups and compared to a fully unconstrained model. In both cases, both ΔCFI and Δχ^2^ indicated no evidence of differences across groups (see Table 2). These null findings suggest generalizability of paths across males and female as well as secondary school- and college-aged youth. 

## 4. Discussion

Our primary aim was to examine the bidirectional associations between positive affect, generosity, entitativity, and intrinsic religiousness across time among adolescents involved in charitable marathon training. Our exploratory hypotheses were partially supported with distinct significant cross-lag paths from pre-training to mid-training (i.e., positive affect → entitativity; interpersonal generosity → positive affect) or from mid-training to post race (i.e., positive affect → interpersonal generosity; interpersonal generosity → fundraising; intrinsic religiousness → interpersonal generosity; intrinsic religiousness → entitativity). Changes in positive affectivity and the internalization of religious ideas were both associated with post-race generosity.

Findings are best interpreted in terms of events that occurred during the course of training and racing. Given that the cross-lag models account for variance explained by previous levels of the same variable, we can interpret significant cross-lag effects from mid-training variables to post race variables as the effects of changes from pre-training to mid-training on post-race outcomes. From pre-training to mid-training participants engaged in heavy training but had not yet achieved their goal of running the race. Participants completed the mid-training assessment the week after the longest training run of the program (half-marathon, 10-mile run; full-marathon, 20-mile run), and participants may have experienced a variety of affective reactions (e.g., anxious about the challenge ahead of them, inspired to complete their goal, excitement about the philanthropic goals of their fundraising). In contrast, participants completed post-race measures the week after completing their race and likely felt the satisfaction of achieving a meaningful goal.

### 4.1. Cross-Lagged Associations of Positive Affectivity on Generosity

We observed a partial upward spiral between generosity and positive affect in the context of charitable marathon training. Young people who reported higher levels of initial generosity felt more positive affect after completing their long run (Time 2), likely because they were in an environment that aligned with their pre-existing tendencies toward prosociality. Positive affect at Time 2 may have then reinforced their generosity after the race. This finding supports previous laboratory-based research that acting generously increases positive affect [29], and positive affect increase generosity [23,24].

However, initial levels of positive affect did not predict mid-training generosity, and mid-training generosity did not predict post-race positive affect, suggesting temporal sequencing matters for character development and the upward spiral theorized by broaden and build theory. Only the increase in positive affectivity from pre- to mid-training rather than initial levels of positive affect (which might be more reflective of trait affectivity) predicted subsequent generosity. In contrast, it may be that people who are dispositionally generous as reflected by initial generosity scores (rather than contextually driven changes in generosity) are more likely to experience positive affect in this environment that emphasizes prosocial action.

### 4.2. Cross-Lagged Effects of Intrinsic Religiousness on Generosity

This study demonstrates that intrinsic religiousness in the context of training for a charity marathon is associated with subsequent generosity across a meaningful sequence of events. Importantly, the analyses demonstrate directionality of effects; the pathway from intrinsic religiousness to subsequent interpersonal generosity was significant, but the pathway from interpersonal generosity to subsequent intrinsic religiousness was not significant. Consistent with theory [43], findings suggest that in the context of charitable marathon training, adolescents’ and emerging adults’ religiousness might be viewed as a unique resource that cultivates positive outcomes, like generosity.

Moreover, the relation between intrinsic religiousness and subsequent generosity was significant even after accounting for variance explained in generosity by previous levels of generosity and positive affectivity (as well as tests for non-significant effects of entitativity and SES). Thus, it is not just the positive affect or group belonging aspects of religion that explain the connection between religiousness and prosociality, as has often been implied in the literature [72]. Instead, the more the participants internalized religious ideas up until mid-training, the more likely they were to report higher levels of generosity (controlling for earlier levels of generosity) at post-race. This supports theories that intrinsic religiousness uniquely influences the development of prosocial qualities [72].

Notably, pre-training levels of intrinsic religiousness did not relate to T2 interpersonal generosity. We might infer that it is more the internalization of the religious ideals of the context rather than dispositional levels of religiousness that matter for generosity change. However, future studies examining the dynamics of change in religiousness and generosity across additional measurement occasions and a greater breadth of time are needed to help clarify the null effect. 

### 4.3. The Role of Entitativity

Contrary to expectations, we did not find a reciprocal association between entitativity and positive affect. Instead, positive affect was only related to subsequent entitativity, which is consistent with the broaden-build theory [23]. Positive affect at the onset of a challenging endeavor like marathon training may facilitate connections with others through the process of training. However, group connection during training does not relate with post-race positive affect. It may be that other unmeasured variables such as race performance satisfaction or feeling of accomplishment are the primary predictors of post-race affect. However, we can only speculate without further data.

Although we did not make specific hypotheses, we explored the cross-lagged relations between entitativity and both generosity and intrinsic religiousness in our model. We found a small negative correlation between mid-training intrinsic religiousness and post-race entitativity. One tentative explanation is that those higher in intrinsic religiousness felt slightly less connected to their training group after the race because they were more connected to traditional religious institutions. Alternately, intrinsic religiousness captures the internal experience of young people that may have shifted the focus of their motivation to complete the race; this may have come at cost of feeling close to the training group. However, because of the lack of theory supporting this finding and small effect size, we are disinclined to make real inferences from it.

In our analyses, we assumed that all TWV training groups would act similarly at a psychological level, but this may have been erroneous. Social network analysis studies previously demonstrated moods and behaviors can have a contagion effect in adolescent groups [73], and we can imagine scenarios in which some TWV groups are more or less inclined to emphasize religiousness, generosity, or positive affect. To truly understand the effects of group closeness across time, group level variables for TWV training groups would need to be included in models to better specify how closeness to the group influences a particular individual nested within a group. Unfortunately, we do not have sufficient statistical power to run the multilevel structural equation models necessary to capture these nesting effects (i.e., far less than the cut-off of 50 level 2 groups typically recommended) [74]. Thus, we recommend caution in interpreting entitativity findings without future replication.

### 4.4. Fundraising and Generosity

We included amount of funds raised as a potential proxy indicator of generosity in our model. Only mid-training interpersonal generosity and SES were significant correlates of amount of money raised for the charitable cause per participant, and small zero-order correlations with funds raised were only significant for T2 generosity and intrinsic religiousness at T1, T2, and T3.

These findings indicate that fundraising may not be a reliable and valid indicator of behavioral generosity. We did not conceptualize it as a direct measure of the amount of time and effort invested in fundraising efforts (e.g., canvassing for donations in the community, raising awareness of the opportunity through social media) but intended it to serve as a concrete proxy for these. However, the wealth of the individuals in participants’ social networks likely affects the success or failure of fundraising efforts. Although analyses controlled for participants’ self-perception of their SES, adolescents are often poor reporters of their SES [75]. Thus, lack of reliability in this measure may mean that we did not properly control for SES. Likewise, a host of unmeasured variables (e.g., extroversion, attractiveness) likely affect fundraising success.

It could also be that self-reported interpersonal generosity and the observed charitable fundraising are not assessing the same type of generosity. The interpersonal generosity scale does not assess the extent to which participants are monetarily generous to others or willing to fundraise; instead, it assesses non-monetary social behaviors within one’s community. In the future, researchers might employ behavioral observation or informant reports of a wider array of participants’ actual generosity expressed in interpersonal relationships (e.g., giving personal funds, making meals for a sick friend) to map a fuller network of generosity related behaviors.

However, it is likely that self-reports of generosity may be biased due to socially desirable responding. There are clear differences between having the desire to be a generous person, viewing oneself as a generous person, and the actual act of fundraising for a charitable cause. Previous research has demonstrated associations between religiousness and socially desirable responding [76,77], so it might be that there is shared variance between change in intrinsic religiousness and self-reported interpersonal generosity in our study. Some have even argued that the religious prosociality hypothesis is a congruence fallacy [78], but others provide evidence that the prosocial effects of religiousness extend beyond socially desirable responding [79,80].

### 4.5. Strengths of the Contextualized Approach

We conducted this research in a unique developmental context, which aligns with a Relational Developmental Systems perspective. All participants were facing a broadly similar experience of training for a marathon, which creates a certain level of parity for participants that would not necessarily exist in other observational studies outside of a laboratory context. Likewise, adolescents and emerging adults regularly and freely engage in athletic training in contexts like those studied in our sample, which provides a level of ecological validity laboratory tasks can never achieve. Finally, longitudinal analyses provide insight into how processes play out in a temporal sequence, suggesting—but not confirming—potential causal effects for future inquiry.

### 4.6. Constraints on Generality

There are of course limitations to this methodology as well. By delving so deeply into the specific population of adolescents and emerging adults who participate in charity marathons, we risk that these findings may not generalize across broader populations. More research on other populations using a similar methodology will be very helpful for determining if these results are generalizable to other athletic contexts. Despite this limitation, the findings demonstrate how athletic training can operate under specific conditions. A related major limitation on generality of this study is the religious particularity of Team World Vision. Though there was some variation in participants’ belief systems, most participants were Christians, and the TWV organization is strongly affiliated with evangelical Christianity. It would be disingenuous for scientists to generalize these findings across other major religious groups without adequately testing for differences. Likewise, although there was some ethnic diversity in the sample (notably with 20% Latino/a), participants were primarily Caucasian, so findings may not generalize across other ethnic groups. Future research should incorporate samples with more diversity in relation to religious, ethnic, and cultural identification.

### 4.7. Other Limitations

In addition to limitations concerning generalizability of the contextual approach, there are several general limitations that should be considered when interpreting these results. First, due to the difficulties of recruiting community-based samples of adolescents, we were unable to collect data from various control groups (e.g., secular charity training team, religious charity without physical training component) that might help to clarify the effects of a specifically religious sporting context. Future studies should collect such naturalistic controls to provide further clarity (though selection effects will always obscure results).

Another limitation is the high rate of attrition for participants in our study, which was mainly due to attrition of the marathon training group. Although participants who completed the study did not differ on the measured study variables, it is possible that there is endogeneity in the sample such that those who dropped out of training did in fact differ on non-measured variables, such as physical health, motivational factors, or other personality constructs. Given these unknowns, we refrain from overgeneralizing the findings without future inquiry into potential selection effects inherent in completing marathon training.

There are also limitations of the cross-lagged approach employed. Although cross-lagged modeling is a strong statistical technique that expands on typical practice in this area of study, it does not model trajectories of change in variables over time like some alternatives (e.g., latent growth curve analysis). However, a cross-lag approach is most appropriate in the present study because it allows examination of transactional processes in a sample evincing relatively stable intra-individual scores across time [81].

Religiousness was measured at the level of intrinsic religiousness, so the focus on intrinsic religiousness may have obscured important but nuanced roles played by other closely related constructs, such as spirituality [82]. However, in a highly religious sample, researchers find it very difficult to differentiate spirituality from religiousness [82]. Likewise, intrinsic religiousness was rather stable across time in study participants, which may explain some of the unexpected cross-lagged effects.

### 4.8. Future Directions

Qualitative methods would be useful at this stage to understand how shifts in identity in a charitable training context relate to the development of generosity and affect. Additionally, quasi-experimental or time series designs that employ some type of control group or waitlist comparisons, though difficult to acquire, would be useful to improve the types of causal inferences that can be made regarding the effects of marathon training.

Future studies might also compare the development of intrinsic religiousness, positive emotions, entitativity, and generosity across time in a variety of religious and non-religious athletic contexts to understand how variable contextual features moderate effects, providing a fuller test of Relational Developmental Systems theory. In the present study, we chose to examine the bidirectional relations among psychosocial variables within the context of religiously affiliated charitable marathon training. This context was chosen because previous research has demonstrated the importance of motivation and social connection in runners [83,84], and there is likely more variability in the extent to which runners experience their training team as entitative. However, it is unknown how effects might vary across different types of sports (e.g., team versus individual) or levels of competition (e.g., elite to recreational). Finally, studies might examine these processes in athletic vs. non-athletic contexts to examine if the embodied nature of athletic involvement leads to amplified moral development as previously hypothesized [85].

## 5. Conclusions

Based on a study design in which adolescents and emerging adults were exposed to a context explicitly supporting prosocial development, our longitudinal cross-lagged model demonstrated the bidirectional associations among positive affect, generosity, and intrinsic religiousness. Participants who reported higher levels of pre-training generosity were more likely to experience positive affect during training, which predicted higher levels of post-race generosity. Likewise, the internalization of religious ideas, reflected in increased intrinsic religiousness during training, was associated with higher post-race generosity. The present evidence does not provide compelling support for the use of fundraising as a behavioral indicator of generosity, but the prediction of post-race fundraising by mid-race generosity suggests further research is warranted.

## Figures and Tables

**Table 1:** Bivariate correlations and descriptive statistics.

	1.	2.	3.	4.	5.	6.	7.	8.	9.	10.	11.	12.	13.	14.
1. Intrinsic Religiousness T1	-													
2. Intrinsic Religiousness T2	0.86 **	-												
3. Intrinsic Religiousness T3	0.85 **	0.86 **	-											
4. Entitativity T1	−0.05	0.02	−0.02	-										
5. Entitativity T2	−0.04	−0.02	−0.02	0.28 **	-									
6. Entitativity T3	−0.06	−0.07	−0.04	0.19 **	0.52 **	-								
7. Pos. Affect T1	0.30 **	0.29 **	0.32 **	0.02	0.08	0.13 *	-							
8. Pos. Affect T2	0.27 **	0.24 **	0.29 **	0.06	0.22 **	0.17 *	0.57 **	-						
9. Pos. Affect T3	0.28 **	0.23 **	0.26 **	0.03	0.21 **	0.25 **	0.58 **	0.68 **	-					
10. Generosity T1	0.30 **	0.24 **	0.27 **	0.05	0.02	0.01	0.29 **	0.31 **	0.22 **	-				
11. Generosity T2	0.27 **	0.25 **	0.24 **	−0.09	0.04	0.05	0.21 **	0.30 **	0.11	0.59 **	-			
12. Generosity T3	0.32 **	0.30 **	0.34 **	−0.02	0.07	0.10	0.21 **	0.30 **	0.29 **	0.67 **	0.50 **	-		
13. Funds raised	0.15 **	0.21 **	0.19 **	−0.01	−0.08	−0.03	0.02	0.00	0.00	0.10	0.15 *	0.08	-	
14. Socioeconomic Status	0.07	0.11	0.100	−0.04	−0.05	−0.11	0.14 **	−0.02	0.03	−0.00	−0.10	−0.08	0.13	-
*M*	4.07	3.98	4.03	3.27	3.82	4.08	3.61	3.50	3.52	5.00	4.90	4.93	1.44	3.88
*SD*	1.02	1.04	1.02	1.64	1.69	1.66	0.86	0.84	0.83	0.63	0.69	0.71	1.20	0.89

Note: T1 = Pre-training. T2 = Mid-training. T3 = Post-race. Intrinsic religiousness and positive affect were scaled from 1 to 5, entitativity, generosity, and wealth were scaled from 1 to 6, and funds raised was scaled from 0 to 4. * *p* < 0.05, ** *p* < 0.01.

**Table 2:** Invariance testing.

	AIC	BIC	χ^2^ (*df*)	SB Δ χ^2^ (*df*)	RMSEA	RMSEA 90% CI	CFI	ΔCFI	Tenable
	**Longitudinal Invariance**								
Null			6230.30 (387) ***						
Configural	14,134.93	14,708.61	324.67 (261) **		0.025	(0.015, 0.033)	0.99		
Metric	14,134.57	14,660.45	345.41 (273) **	20.02 (12)	0.026	(0.016, 0.034)	0.99	0.001	Yes
Scalar	14,131.50	14,609.57	366.27 (285) ***	21.10 (12)	0.027	(0.018, 0.034)	0.99	0.002	Yes
	**Invariance by Age**								
Null			7871.20 (1096) ***						
Configural	15,905.16	17,047.11	1252.83 (824) ***		0.053	(0.047, 0.059)	0.94		
Metric	15,905.85	17,024.34	1265.03 (830) ***	12.20 (6)	0.053	(0.047, 0.059)	0.94	0.001	Yes
Scalar	15,922.81	16,994.37	1305.89 (842) ***	40.86 (12) ***	0.055	(0.049, 0.060)	0.93	0.004	Yes
Structural	15,892.84	16,850.99	1328.83 (871) ***	22.94 (29)	0.053	(0.048, 0.059)	0.93	−0.001	Yes
	**Invariance by Gender**								
Null			7770.3 (1096) ***						
Configural	17,082.25	18,234.15	1325.69 (825) ***		0.056	(0.050, 0.062)	0.92		
Metric	17,079.54	18,203.74	1334.28 (832) ***	9.15 (7)	0.056	(0.050, 0.0610	0.92	0.000	Yes
Scalar	17,084.99	18,161.68	1364.28 (844) ***	29.77 (12) **	0.056	(0.051, 0.062)	0.92	0.003	Yes
Structural	17,061.57	18,023.47	1390.94 (873) ***	29.87 (29)	0.055	(0.050, 0.061)	0.92	0.000	Yes

Note. The null model for longitudinal data was estimated based on recommendations from Little (2013), such that item means and variances were estimated and constrained to equality over time and covariances were set to zero. Maximum likelihood (ML) estimation was used. AIC = Akaike Information Criterion, BIC = Bayesian Information Criterion, SB = Satorra–Bentler, RMSEA = Root Mean Squared Error of Approximation, CFI = Comparative Fit Index. * *p* < 0.05, ** *p* < 0.01, *** *p* < 0.001.

## Supplementary Materials

The following are available online at https://www.mdpi.com/1660-4601/17/3/686/s1, Table S1: Full results from final autoregressive models, File S1: Syntax for Autoregressive Models, File S2: Complete List of Measures.
