# Supplementary material for: Bidirectional Associations across Time between Entitativity, Positive Affect, Generosity, and Religiousness in Adolescents Training with a Religiously Affiliated Charity Marathon Team
Source: Int J Environ Res Public Health. 2020 Jan 21;17(3):686. doi: 10.3390/ijerph17030686 (PMC7037786; doi:10.3390/ijerph17030686)
Supplement: Supplementary file 1 [file ijerph-17-00686-s001.pdf]

Online Supplement Materials to Accompany

Supplemental Table 1. Full results from final autoregressive models.

	Intrinsic Religiousness	Entitativity	Positive Affectivity	Interpersonal Generosity	Fundraising
**Regressed on T2**					
Intrinsic Religiousness	0.91, p < .001	-0.12, p = .049	0.05, p = .479	0.17, p = .016	0.08, p = .174
Entitativity	-0.03, p = .481	0.50, p < .001	0.01, p = .862	0.03, p = .564	-0.11, p = .133
Positive Affectivity	0.08, p = .088	0.11, p = .164	0.78, p < .001	0.19, p = .053	-0.06, p = .453
Interpersonal Generosity	0.05, p = .307	0.02, p = .704	-0.08, p = .222	0.46, p < .001	0.19, p = .016
SES	-	-	-	-	0.14, p = .008
**Regressed on T1**					
Intrinsic Religiousness	0.93, p < .001	-0.09, p = .161	0.08, p = .239	0.01, p = .108	
Entitativity	0.05, p = .129	0.31, p < .001	0.06, p = .277	-0.05, p = .453	
Positive Affectivity	0.01, p = .745	0.15, p = .053	0.59, p < .001	0.00, p = .948	
Interpersonal Generosity	-0.03, p = .482	0.04, p = .603	0.19, p = .018	0.64, p < .001	
SES	0.08, p = .208	-0.04, p = .423	0.15, p = .01	-0.01, p = .831	

* Note. Confidence intervals are not available in M*plus* when using full information maximum likelihood estimation with robust standard errors. .

Syntax for Autoregressive Models

Title: Full Model

```
DATA: File is mplustake2.dat;
Variable: NAMES ARE ExternalDataReference sample
T1there T2there T3there T4there
Times T1weeks T2weeks T3weeks T4weeks T1weeksprop
T2weeksprop T3weeksprop T4weeksprop T1_2weeks T2_3weeks T3_4weeks
TIME1 Finished Age Gender Eth RelTrad
BelGod Grade Fathwork Mothwork Relation Edmom
Eddad Wealth Moves STI1 STI2 STI3
STI4 STI5 STI6 STI7 STI8 DUREL1
DUREL2 DUREL3 DUREL4 DUREL5 GCQ1 GCQ2
GCQ3 GCQ4 GCQ5 SC1 SC2 SC3
SC4 SC5 SC6 SC7 SC8 SC9
SC10 SC11 SC12 SC13 SC14 SC15
SC16 SC17 SC18 SC19 SC20 SC21
SC22 SC23 SC24 SC25 SC26 SC27
SC28 SC29 SC30 SC31 SC32 SC33
SC34 SC35 SC36 PQ1 PQ2 PQ3
PQ4 PQ5 PQ6 PQ7 PQ8 PQ9
PQ10 PQ11 GRB1 GRB2 GRB3 GRB4
GRB5 GRB6 GRB7 GRB8 GRB9 GRB10
GRB11 GRB12 GRB13 GRB14 GRB15 GRB16
ERQ1 ERQ2 ERQ3 ERQ4 ERQ5 ERQ6
ERQ7 ERQ8 ERQ9 ERQ10 ERQ11 ERQ12
ERQ13 ERQ14 ERQ15 IGS1 IGS2 IGS3
IGS4 IGS5 IGS6 IGS7 IGS8 IGS9
IGS10 SWLS1 SWLS2 SWLS3 SWLS4 SWLS5
YRBSS1 YRBSS2 YRBSS3 YRBSS4 YRBSS5 YRBSS6
YRBSS7 YRBSS8 YRBSS9 YRBSS10 YRBSS11 YRBSS12
YRBSS13 YRBSS14 YRBSS15 CSW1 CSW2 CSW3
CSW4 CSW5 CSW6 CSW7 CSW8 CSW9
CSW10 CSW11 CSW12 CSW13 CSW14 CSW15
PANAS1 PANAS2 PANAS3 PANAS4 PANAS5 PANAS6
PANAS7 PANAS8 PANAS9 PANAS10 GE1_T1 MTTWV1
MTTWV2 MTTWV3 saddys sadreg angdys angreg
posaff_T1 negaff_T1 compcsw godcsw valcsw SWLS_Score
PQ7r PQ10r PQ_Interpersonal PQ_LifeHardship PQ_DailyHassles PQ_Total
SC2r SC3r SC4r SC6r SC8r SC9r
SC10r SC11r SC12r SC14r SC16r SC17r
SC19r SC20r SC21r SC23r SC25r SC28r
SC29r SC31r SC32r SC33r SC34r SC35r
SC intgen_T1 yrbtob yrbss10r yrbss13r yrbss14r
yrbdiet yrbphysact GRB3R GRB4R GRB5R GRB7R
GRB15R GRBTOT IR_T1 STI_tot Gwatch TIME2
Finished_T2 DUREL1_T2 DUREL2_T2 DUREL3_T2 DUREL4_T2 DUREL5_T2
STI1_T2 STI2_T2 STI3_T2 STI4_T2 STI5_T2 STI6_T2
STI7_T2 STI8_T2 GCQ1_T2 GCQ2_T2 GCQ3_T2 GCQ4_T2
GCQ5_T2 SC1_T2 SC2_T2 SC3_T2 SC4_T2 SC5_T2
SC6_T2 SC7_T2 SC8_T2 SC9_T2 SC10_T2 SC11_T2
SC12_T2 SC13_T2 SC14_T2 SC15_T2 SC16_T2 SC17_T2
```

SC18_T2 SC19_T2 SC20_T2 SC21_T2 SC22_T2 SC23_T2
SC24_T2 SC25_T2 SC26_T2 SC27_T2 SC28_T2 SC29_T2
SC30_T2 SC31_T2 SC32_T2 SC33_T2 SC34_T2 SC35_T2
SC36_T2 PQ1_T2 PQ2_T2 PQ3_T2 PQ4_T2 PQ5_T2
PQ6_T2 PQ7_T2 PQ8_T2 PQ9_T2 PQ10_T2 PQ11_T2
GRB1_T2 GRB2_T2 GRB3_T2 GRB4_T2 GRB5_T2 GRB6_T2
GRB7_T2 GRB8_T2 GRB9_T2 GRB10_T2 GRB11_T2 GRB12_T2
GRB13_T2 GRB14_T2 GRB15_T2 GRB16_T2 ERQ1_T2 ERQ2_T2
ERQ3_T2 ERQ4_T2 ERQ5_T2 ERQ6_T2 ERQ7_T2 ERQ8_T2
ERQ9_T2 ERQ10_T2 ERQ11_T2 ERQ12_T2 ERQ13_T2 ERQ14_T2
ERQ15_T2 IGS1_T2 IGS2_T2 IGS3_T2 IGS4_T2 IGS5_T2
IGS6_T2 IGS7_T2 IGS8_T2 IGS9_T2 IGS10_T2 SWLS1_T2
SWLS2_T2 SWLS3_T2 SWLS4_T2 SWLS5_T2 YRBSS1_T2 YRBSS2_T2
YRBSS3_T2 YRBSS4_T2 YRBSS5_T2 YRBSS6_T2 YRBSS7_T2 YRBSS8_T2
YRBSS9_T2 YRBSS10_T2 YRBSS11_T2 YRBSS12_T2 YRBSS13_T2 YRBSS14_T2
YRBSS15_T2 CSW1_T2 CSW2_T2 CSW3_T2 CSW4_T2 CSW5_T2
CSW6_T2 CSW7_T2 CSW8_T2 CSW9_T2 CSW10_T2 CSW11_T2
CSW12_T2 CSW13_T2 CSW14_T2 CSW15_T2 PANAS1_T2 PANAS2_T2
PANAS3_T2 PANAS4_T2 PANAS5_T2 PANAS6_T2 PANAS7_T2 PANAS8_T2
PANAS9_T2 PANAS10_T2 GE1_T2 PrevRace_T2 PrevRaceNum_T2 PrevRaceType_T2
MTTWV1_T2 MTTWV2_T2 MTTWV3_T2 saddys_T2 sadreg_T2 angdys_T2
angreg_T2 posaff_T2 negaff_T2 compcsw_T2 godcsw_T2 valcsw_T2
SWLS_Score_T2 PQ7r_T2 PQ10r_T2 PQ_Interpersonal_T2
PQ_LifeHardship_T2 PQ_DailyHassles_T2
PQ_Total_T2 SC2r_T2 SC3r_T2 SC4r_T2 SC6r_T2 SC8r_T2
SC9r_T2 SC10r_T2 SC11r_T2 SC12r_T2 SC14r_T2 SC16r_T2
SC17r_T2 SC19r_T2 SC20r_T2 SC21r_T2 SC23r_T2 SC25r_T2
SC28r_T2 SC29r_T2 SC31r_T2 SC32r_T2 SC33r_T2 SC34r_T2
SC35r_T2 SC_T2 intgen_T2 yrbtob_T2 yrbss10r_T2 yrbss13r_T2
yrbss14r_T2 yrbdiet_T2 yrbphysact_T2 GRB3R_T2 GRB4R_T2 GRB5R_T2
GRB7R_T2 GRB15R_T2 GRBTOT_T2 IR_T2 STI_tot_T2 Gwatch_T2
TIME3 Finished_T3 DUREL1_T3 DUREL2_T3 DUREL3_T3 DUREL4_T3
DUREL5_T3 STI1_T3 STI2_T3 STI3_T3 STI4_T3 STI5_T3
STI6_T3 STI7_T3 STI8_T3 GCQ1_T3 GCQ2_T3 GCQ3_T3
GCQ4_T3 GCQ5_T3 SC1_T3 SC2_T3 SC3_T3 SC4_T3
SC5_T3 SC6_T3 SC7_T3 SC8_T3 SC9_T3 SC10_T3
SC11_T3 SC12_T3 SC13_T3 SC14_T3 SC15_T3 SC16_T3
SC17_T3 SC18_T3 SC19_T3 SC20_T3 SC21_T3 SC22_T3
SC23_T3 SC24_T3 SC25_T3 SC26_T3 SC27_T3 SC28_T3
SC29_T3 SC30_T3 SC31_T3 SC32_T3 SC33_T3 SC34_T3
SC35_T3 SC36_T3 PQ1_T3 PQ2_T3 PQ3_T3 PQ4_T3
PQ5_T3 PQ6_T3 PQ7_T3 PQ8_T3 PQ9_T3 PQ10_T3
PQ11_T3 GRB1_T3 GRB2_T3 GRB3_T3 GRB4_T3 GRB5_T3
GRB6_T3 GRB7_T3 GRB8_T3 GRB9_T3 GRB10_T3 GRB11_T3
GRB12_T3 GRB13_T3 GRB14_T3 GRB15_T3 GRB16_T3 ERQ1_T3
ERQ2_T3 ERQ3_T3 ERQ4_T3 ERQ5_T3 ERQ6_T3 ERQ7_T3
ERQ8_T3 ERQ9_T3 ERQ10_T3 ERQ11_T3 ERQ12_T3 ERQ13_T3
ERQ14_T3 ERQ15_T3 IGS1_T3 IGS2_T3 IGS3_T3 IGS4_T3
IGS5_T3 IGS6_T3 IGS7_T3 IGS8_T3 IGS9_T3 IGS10_T3
SWLS1_T3 SWLS2_T3 SWLS3_T3 SWLS4_T3 SWLS5_T3 YRBSS1_T3
YRBSS2_T3 YRBSS3_T3 YRBSS4_T3 YRBSS5_T3 YRBSS6_T3 YRBSS7_T3
YRBSS8_T3 YRBSS9_T3 YRBSS10_T3 YRBSS11_T3 YRBSS12_T3 YRBSS13_T3

YRBSS14_T3 YRBSS15_T3 CSW1_T3 CSW2_T3 CSW3_T3 CSW4_T3
CSW5_T3 CSW6_T3 CSW7_T3 CSW8_T3 CSW9_T3 CSW10_T3
CSW11_T3 CSW12_T3 CSW13_T3 CSW14_T3 CSW15_T3 PANAS1_T3
PANAS2_T3 PANAS3_T3 PANAS4_T3 PANAS5_T3 PANAS6_T3 PANAS7_T3
PANAS8_T3 PANAS9_T3 PANAS10_T3 GE1_T3 PrevRace_T3 PrevRaceNum_T3
PrevRaceType_T3 MTTWV1_T3 MTTWV2_T3 MTTWV3_T3 saddys_T3 sadreg_T3
angdys_T3 angreg_T3 posaff_T3 negaff_T3 compcsw_T3 godcsw_T3
valcsw_T3 SWLS_Score_T3 PQ7r_T3 PQ10r_T3 PQ_Interpersonal_T3
PQ_LifeHardship_T3
PQ_DailyHassles_T3 PQ_Total_T3 SC2r_T3 SC3r_T3 SC4r_T3 SC6r_T3
SC8r_T3 SC9r_T3 SC10r_T3 SC11r_T3 SC12r_T3 SC14r_T3
SC16r_T3 SC17r_T3 SC19r_T3 SC20r_T3 SC21r_T3 SC23r_T3
SC25r_T3 SC28r_T3 SC29r_T3 SC31r_T3 SC32r_T3 SC33r_T3
SC34r_T3 SC35r_T3 SC_T3 intgen_T3 yrbtob_T3 yrbss10r_T3
yrbss13r_T3 yrbss14r_T3 yrbdiet_T3 yrbphysact_T3 GRB3R_T3 GRB4R_T3
GRB5R_T3 GRB7R_T3 GRB15R_T3 GRBTOT_T3 IR_T3 STI_tot_T3
Gwatch_T3 RanRace TIME4 Finished_T4 DUREL_T4 DUREL1_T4
DUREL2_T4 DUREL3_T4 DUREL4_T4 DUREL5_T4 STI1_T4 STI2_T4
STI3_T4 STI4_T4 STI5_T4 STI6_T4 STI7_T4 STI8_T4
GCQ1_T4 GCQ2_T4 GCQ3_T4 GCQ4_T4 GCQ5_T4 SC1_T4
SC2_T4 SC3_T4 SC4_T4 SC5_T4 SC6_T4 SC7_T4
SC8_T4 SC9_T4 SC10_T4 SC11_T4 SC12_T4 SC13_T4
SC14_T4 SC15_T4 SC16_T4 SC17_T4 SC18_T4 SC19_T4
SC20_T4 SC21_T4 SC22_T4 SC23_T4 SC24_T4 SC25_T4
SC26_T4 SC27_T4 SC28_T4 SC29_T4 SC30_T4 SC31_T4
SC32_T4 SC33_T4 SC34_T4 SC35_T4 SC36_T4 PQ1_T4
PQ2_T4 PQ3_T4 PQ4_T4 PQ5_T4 PQ6_T4 PQ7_T4
PQ8_T4 PQ9_T4 PQ10_T4 PQ11_T4 GRB1_T4 GRB2_T4
GRB3_T4 GRB4_T4 GRB5_T4 GRB6_T4 GRB7_T4 GRB8_T4
GRB9_T4 GRB10_T4 GRB11_T4 GRB12_T4 GRB13_T4 GRB14_T4
GRB15_T4 GRB16_T4 ERQ1_T4 ERQ2_T4 ERQ3_T4 ERQ4_T4
ERQ5_T4 ERQ6_T4 ERQ7_T4 ERQ8_T4 ERQ9_T4 ERQ10_T4
ERQ11_T4 ERQ12_T4 ERQ13_T4 ERQ14_T4 ERQ15_T4 IG1_T4
IG2_T4 IG3_T4 IG4_T4 IG5_T4 IG6_T4 IG7_T4
IG8_T4 IG9_T4 IG10_T4 SWLS1_T4 SWLS2_T4 SWLS3_T4
SWLS4_T4 SWLS5_T4 YRBSS1_T4 YRBSS2_T4 YRBSS3_T4 YRBSS4_T4
YRBSS5_T4 YRBSS6_T4 YRBSS7_T4 YRBSS8_T4 YRBSS9_T4 YRBSS10_T4
YRBSS11_T4 YRBSS12_T4 YRBSS13_T4 YRBSS14_T4 YRBSS15_T4 CSW1_T4
CSW2_T4 CSW3_T4 CSW4_T4 CSW5_T4 CSW6_T4 CSW7_T4
CSW8_T4 CSW9_T4 CSW10_T4 CSW11_T4 CSW12_T4 CSW13_T4
CSW14_T4 CSW15_T4 PANAS1_T4 PANAS2_T4 PANAS3_T4 PANAS4_T4
PANAS5_T4 PANAS6_T4 PANAS7_T4 PANAS8_T4 PANAS9_T4 PANAS10_T4
GE2_T4 MT3401_T4 MT3402_T4 MT3403_T4 TOA2FU6_T4 TOA2FU7_T4
saddys_T4 sadreg_T4 angdys_T4 angreg_T4 posaff_T4 negaff_T4
compcsw_T4 godcsw_T4 valcsw_T4 SWLS_Score_T4 PQ7r_T4 PQ10r_T4
PQ_Interpersonal_T4 PQ_LifeHardship_T4 PQ_DailyHassles_T4
PQ_Total_T4 SC2r_T4 SC3r_T4
SC4r_T4 SC6r_T4 SC8r_T4 SC9r_T4 SC10r_T4 SC11r_T4
SC12r_T4 SC14r_T4 SC16r_T4 SC17r_T4 SC19r_T4 SC20r_T4
SC21r_T4 SC23r_T4 SC25r_T4 SC28r_T4 SC29r_T4 SC31r_T4
SC32r_T4 SC33r_T4 SC34r_T4 SC35r_T4 SC_T4 SC_T4c
intgen_T4 PQTot_T4 PQ_Int_T4 PQ_LH_T4 PQ_DH_T4 yrbtob_T4

```
  yrbss10r_T4 yrbss13r_T4 yrbss14r_T4 yrbdiet_T4 yrbphysact_T4 GRB3R_T4
  GRB4R_T4 GRB5R_T4 GRB7R_T4 GRB15R_T4 GRBTOT_T4 IR_T4
  STI_tot_T4 Gwatch_T4 GROUPENTITATIVITY GE_cl_t1 GE_centeredness GE_cl_T2
  GE_centeredness_T2 GE_cl_T3 GE_centeredness_T3 GE_CLOSENESS_T4
  GE_CENTEREDNESS_T4 TRAINING
  Finished_L1 TrainingLog_L1 TL1_L1 TL2_L1 TL3_L1 TL4_L1
  TL5_L1 TL6_L1 TL7_L1 Finished_L2 TrainingLog_L2 TL1_L2
  TL2_L2 TL3_L2 TL4_L2 TL5_L2 TL6_L2 TL7_L2
  Finished_L3 TrainingLog_L3 TL1_L3 TL2_L3 TL3_L3 TL4_L3
  TL5_L3 TL6_L3 TL7_L3 Finished_L4 TrainingLog_L4 TL1_L4
  TL2_L4 TL3_L4 TL4_L4 TL5_L4 TL6_L4 TL7_L4
  FUNDING FundTot FundNum tmotiv tclose Emreg_T1
  Emreg_T2 Emreg_T3 Emreg_T4 GRBTOT_T1 Phi_T1 Phi_T2
  Phi_T3 Phi_T4 Emregr_t1 Emregr_t2 Emregr_t3 Emregr_t4
  sadregr_t1 sadregr_t2 sadregr_t3 sadregr_t4 SC2_T2R SC3_T2R
  SC4_T2R SC6_T2R SC8_T2R SC9_T2R SC10_T2R SC11_T2R
  SC12_T2R SC14_T2R SC16_T2R SC17_T2R SC19_T2R SC20_T2R
  SC21_T2R SC23_T2R SC25_T2R SC28_T2R SC29_T2R SC31_T2R
  SC32_T2R SC33_T2R SC34_T2R SC35_T2R SC2_T3R SC3_T3R
  SC4_T3R SC6_T3R SC8_T3R SC9_T3R SC10_T3R SC11_T3R
  SC12_T3R SC14_T3R SC16_T3R SC17_T3R SC19_T3R SC20_T3R
  SC21_T3R SC23_T3R SC25_T3R SC28_T3R SC29_T3R SC31_T3R
  SC32_T3R SC33_T3R SC34_T3R SC35_T3R SC2_T4R SC3_T4R
  SC4_T4R SC6_T4R SC8_T4R SC9_T4R SC10_T4R SC11_T4R
  SC12_T4R SC14_T4R SC16_T4R SC17_T4R SC19_T4R SC20_T4R
  SC21_T4R SC23_T4R SC25_T4R SC28_T4R SC29_T4R SC31_T4R
  SC32_T4R SC33_T4R SC34_T4R SC35_T4R FUNDNEW INTGN_T1
  INTGN_T2 INTGN_T3 AgeGr;


    USEVariables ARE
PANAS10
PANAS10_T2
PANAS10_T3
GE_cl_T1 GE_cl_T2 GE_cl_T3
FUNDNEW wealth
IGP1 IGP2 IGP3
IGP1_T2 IGP2_T2 IGP3_T2
IGP1_T3 IGP2_T3 IGP3_T3
PAP2 PAP1 PAP2_T2 PAP1_T2
PAP2_T3 PAP1_T3
REL3R REL4R REL5R
REL3R_T2 REL4R_T2 REL5R_T2
REL3R_T3 REL4R_T3 REL5R_T3;

    MISSING ARE ALL (-999);

Define:
IGP1=(IGS5+IGS10+IGS9+IGS3)/4;
IGP2=(IGS6+IGS8+IGS2)/3;
IGP3=(IGS7+IGS4+IGS1)/3;
IGP1_T2=(IGS5_T2+IGS10_T2+IGS9_T2+IGS3_T2)/4;
```

```
IGP2_T2=(IGS6_T2+IGS8_T2+IGS2_T2)/3;
IGP3_T2=(IGS7_T2+IGS4_T2+IGS1_T2)/3;
IGP1_T3=(IGS5_T3+IGS10_T3+IGS9_T3+IGS3_T3)/4;
IGP2_T3=(IGS6_T3+IGS8_T3+IGS2_T3)/3;
IGP3_T3=(IGS7_T3+IGS4_T3+IGS1_T3)/3;
PAP2=(PANAS2+PANAS8)/2;
PAP1=(PANAS6+PANAS5)/2;
PAP2_T2=(PANAS2_T2+PANAS8_T2)/2;
PAP1_T2=(PANAS6_T2+PANAS5_T2)/2;
PAP2_T3=(PANAS2_T3+PANAS8_T3)/2;
PAP1_T3=(PANAS6_T3+PANAS5_T3)/2;
REL3R=6-DUREL3;
REL4R=6-DUREL4;
REL5R=6-DUREL5;
REL3R_T2=6-DUREL3_T2;
REL4R_T2=6-DUREL4_T2;
REL5R_T2=6-DUREL5_T2;
REL3R_T3=6-DUREL3_T3;
REL4R_T3=6-DUREL4_T3;
REL5R_T3=6-DUREL5_T3;
Analysis:
estimator = mlr;

  MODEL:
!Latent Variables
InterT1 by IGP1(1)
IGP2(2)
IGP3(3);
PAT1 by PAP1(4)
PAP2(5)
PANAS10(6);
IntRlgT1 by REL3R(7)
REL4R(8)
REL5R(9);
InterT2 by IGP1_T2(1)
IGP2_T2(2)
IGP3_T2(3);
PAT2 by PAP2_T2(4)
PAP1_T2(5)
PANAS10_T2(6);
IntRlgT2 by REL3R_T2(7)
REL4R_T2(8)
REL5R_T2(9);
InterT3 by IGP1_T3(1)
IGP2_T3(2)
IGP3_T3(3);

PAT3 by PAP2_T3(4)
PAP1_T3(5)
PANAS10_T3(6);
IntRlgT3 by REL3R_T3(7)
REL4R_T3(8)
```

```
REL5R_T3(9);

!Correlate items across waves
IGP1 WITH IGP1_T2 IGP1_T3;
IGP1_T2 WITH IGP1_T3;
IGP2 WITH IGP2_T2 IGP2_T3;
IGP2_T2 WITH IGP2_T3;
IGP3 WITH IGP3_T2 IGP3_T3;
IGP3_T2 WITH IGP3_T3;
PAP2_T3 with PAP2_T2 PAP2;
PAP2_T2 WITH PAP2;
PAP1_T3 with PAP1_T2 PAP1;
PAP1_T2 WITH PAP1;
PANAS10 with PANAS10_T2 PANAS10_T3;
PANAS10_T2 WITH PANAS10_T3;
REL3R_T3 WITH REL3R_T2 REL3R;
REL3R_T2 WITH REL3R;
REL4R_T3 WITH REL4R_T2 REL4R;
REL4R_T2 WITH REL4R;
REL5R_T3 WITH REL5R_T2 REL5R;
REL5R_T2 WITH REL5R;

[InterT1@0  PAT1@0 IntRlgT1@0 InterT2 PAT2 IntRlgT2 InterT3 PAT3 IntRlgT3];

[PANAS10 PANAS10_T2 PANAS10_T3](10);
[IGP1 IGP1_T2 IGP1_T3](11);
[IGP2 IGP2_T2 IGP2_T3](12);
[IGP3 IGP3_T2 IGP3_T3](13);
[PAP1 PAP1_T2 PAP1_T3](14);
[PAP2 PAP2_T2 PAP2_T3](15);
[REL3R REL3R_T2 REL3R_T3](16);
[REL4R REL4R_T2 REL4R_T3](17);
[REL5R REL5R_T2 REL5R_T3](18);


!Autoregressive paths
InterT3 on InterT2;
PAT3 on pat2;
IntRlgT3 on IntRlgT2;
InterT2 on InterT1;
PAT2 on pat1;
IntRlgT2 on IntRlgT1;
GE_cl_T2 on GE_cl_T1;
GE_cl_T3 on GE_cl_T2 ;

!Cross-Lagged paths
InterT3 on PAT2
IntRlgT2
GE_cl_T2;
PAT3 on InterT2
IntRlgT2
GE_cl_T2;
```

```
IntRlgT3 on InterT2
PAT2
GE_cl_T2;
GE_cl_T3 on InterT2
PAT2
IntRlgT2;
InterT2 on PAT1
IntRlgT1
GE_cl_T1;
PAT2 on InterT1
IntRlgT1
GE_cl_T1;
IntRlgT2 on InterT1
PAT1
GE_cl_T1;
GE_cl_T2 on InterT1
PAT1
IntRlgT1;

!Fundraising outcome path
fundnew on InterT2
PAT2
IntRlgT2
GE_cl_T2
wealth;

!Allow correlation with all DVs
GE_cl_T3 with InterT3 PAT3 IntRlgT3;
GE_cl_T2 with InterT2 PAT2 IntRlgT2;
GE_cl_T1 with InterT1 PAT1 IntRlgT3;

!Covariate paths
PAT1 on wealth;
InterT1 on wealth;
IntRlgT1 on wealth;
GE_cl_T1 on wealth;

Output: STDYX MODindices (3.84)
```

Complete List of Measures

Self-Control Scale. Tangney, Baumeister, & Boone's (2004) scale contains items measuring self-control (e.g., "I am good at resisting temptation") on a 1 (Not at All) to 5 (Very Much) scale. We will use the abbreviated, 13-item version of the scale. (Items included in the brief version are marked with an asterisk in the appendix.)

3-Factor Patience Questionnaire. The 11-item 3-Factor Patience Questionnaire (3-FPQ; Schnitker, 2012) will be used as a measure of interpersonal ("My friends would say I'm a very patient friend"), life hardships ("I find it pretty easy to be patient with a difficult life problem or illness"), and daily hassles ("Although they're annoying, I don't get too upset when stuck in a traffic jam") patience. Items are rated from 1 = Not like me at all to 5 = Very Much Like Me.

General Regulatory Behavior Questionnaire. A questionnaire was developed based on Oaten and Cheng's (2004) study items measuring everyday regulatory behaviors, including cigarette smoking, alcohol and caffeine consumptions, dietary habits, self-care habits, spending habits, emotion control, study habits, obeying rules, use of social media, energy conservation, and turning in assignments. Participants were asked to rate how often they engaged in behaviors in the past week on a scale from 9 (not at all) to 5 (almost always).

Emotion Regulation. Emotion regulation of participants will be assessed using three subscales (i.e., 4-item anger regulation coping, 4-item emotion regulation inhibition, and 3-item dysregulated expression) from the Children's Sadness and Anger Management Scale (Zeman, Shipman, & Penza-Clyve, 2001. Participants will report on sadness and anger. A sample item follows: "When I am feeling mad, I control my temper" (anger/sadness regulation coping), " I hide my anger/sadness" (inhibition), and "I attack whatever it is that makes me mad." (dysregulated coping). The response choices follow: 0 = "not true," 1 = "somewhat true," 2 = "very true".

Interpersonal Generosity Scale. The 10-item Interpersonal Generosity scale (Smith, & Hill, 2009) will be used as a measure of generosity. The items measure six identifiable dimensions of interpersonal generosity: attention ("I am known by family and friends as someone who makes time to pay attention to others' problems"), compassion ("When friends or family members experience something upsetting or discouraging, I make a special point of being kind to them"), open-handedness ("When it comes to my personal relationships with others, I am a very generous person"), self-extension ("My decisions are often based on concern for the welfare of others"), courage ("I am usually willing to risk my own feelings being hurt in the process if I stand a chance of helping someone else in need"), and verbal expression ("I make it a point to let my friends and family know how much I love and appreciate them") on a 1 (Strongly Disagree) to 6 (Strongly Agree) scale.

Duke University Religious Index (DUREL). The Duke University Religion Index (DUREL; Koenig & Bussing, 2010) is a five-item measure of religious involvement that includes dimensions of religiosity (organizational religious activity, non-organizational religious activity, and intrinsic religiosity). Organizational religious activity (ORA; How often do you attend church or other religious meetings?) and non-organizational religious activity (NORA; How often do you spend time in private religious activities, such as prayer, meditation or Bible?) are both rated on a 6-point scale. For ORA the response choice is as follows: 1 - Never; 2 - Once a year or less; 3 - A few times a year; 4 - A few times a month; 5 - Once a week; 6 - More than once/week; while, NORA includes: 1 - Rarely or never; 2 - A few times a month; 3 - Once a week; 4 - Two or more times/week; 5 - Daily; 6 - More than once a day. Intrinsic religiosity is measured based on three items (e.g. In my life, I experience the presence of the Divine (i.e., God) and the response choices are 1 - Definitely not true; 2 - Tends not to be true; 3 - Unsure; 4 - Tends to be true; 5 - Definitely true of me.

Spiritual Transcendence Index. The 8-item Spiritual Transcendence Index (STI; Seidlitz et. al, 2002) measured the meaning and quality of a participant's spirituality using a 6-point Likert scale (1 = Strongly disagree to 6 = Strongly agree) on items such as, "Maintaining my spirituality is a priority for me."

God Concept Questions. Based on Laurin, Kay, and Fitzsimons (2012), perceptions of God's sovereignty and watchfulness. Perceptions of God's sovereignty will be rated from 1 to 5 based on the degree to which the person conceives of God as in control of their success or failure in life. "My

future success in life depends...(1) completely on factors God controls (2) mostly on factors that God controls (3) equally on factors that God and I control (4) mostly on factors that I control (5) completely on factors I control. Perceptions of God watching will be rated from 1 to 5 on a Likert scale. "If God (or some non-human spiritual being) exists, it is likely that God watches peoples' behavior and notices when they misbehave." Additionally, the monitoring by God subscale (3 items) based on Carter, McCullough & Carver (2012) will also be used. Ratings will be based on a 1 (Not at all) to 7 (Very true) scale and include items such as "I believe a higher power can see my behavior."

Satisfaction with Life Scale. This 5-item scale (Diener, Emmons, Larsen, & Griffin, 1985) measures global satisfaction with life. Items such as "In most ways, my life is close to my ideal" are rated on a scale from 1 (strongly disagree) to 7 (strongly agree).

Youth Risk Behavior Surveillance. Participants will be administered 16 items selected from Youth Risk Behavior Surveillance measure utilized by Center for Disease control (Foti, Balaji, & Shanklin, 2011). Items to be administered relate to tobacco, alcohol, and marijuana use, dietary habits, exercise activities, safe driving, and media/technology use.

Positive and Negative Affect Schedule. The shortened version of the PANAS-C (Ebesutani et al., 2012; Laurent, et al. 1999) will be used that includes 5-item PA scale (joyful, cheerful, happy, lively, proud) and a 5-item NA scale (miserable, mad, afraid, scared, sad) rated on a 1 (very slightly or not at all) to 5 (extremely) scale.

Group Entitativity Measure. The GEM-in (Gaertner & Schopler, 1998) measures perceived interconnections between self and others. Test-takers choose between 6 different pictures of the self and others (pictorially represented by circles). The first picture has the greatest distance between self and others, while the last picture shows no distance between self and others, so that the group and self are completely overlapping. Pictures at each of the 3 levels of distance also manipulated whether the circle representing the self was seen as central vs. periphery to the group. Participants will be instructed to complete the measure relative to their athletic teams/extracurricular activity group (alternative instructions).

Performances and Motivation Orientation. To assess participants instrumental, moral, and spiritual motivations for training, we will ask them to rate the extent to which they are motivated to train with Team World Vision because of opportunities to improve physical fitness, raise money for clean water, and grow spiritually. At later time points, we will ask participants the extent to which they change in each of these domains as a result of training. Ratings will be made on a 1 (Not at all true of me) to 5 (Always true for me) scale.

Contingencies of Self-worth. Further, participants will report on 3 subscales of contingencies of self worth rated on a 1 (Strongly disagree) to 7 (Strongly agree) and each subscale has 5 items (Crocker et al., 2003; competition, virtue, God's love). Participants will rate Competition contingencies will include statements such as "my self-worth is affected by how well I do when I am competing with others". Virtue contingencies includes statements like "I couldn't respect myself if I didn't live up to a moral code" and "When I think that I am disobeying God, I feel bad about myself" for God's love contingencies.

Fundraising and training outcomes. Data will be collected directly from Team World Vision documenting participant fundraising. To assess sponsorship trends, participants will note their involvement in sponsorships programs before and after training for either the half or full marathon. Participants will also be asked to note how frequently they participated in TWV group runs.

Demographics.
